# Association between parenting styles and weight-for-length z scores among infants and toddlers aged 0–36 months in China: a cross-sectional study

**DOI:** 10.3389/fped.2026.1827938

**Published:** 2026-05-20

**Authors:** Jing Sun, Min Yu, Haiyan He, Min Zhu, Anhui Zhang

**Affiliations:** Department of Child Health Care, Wuhu Maternal and Child Health and Family Planning Service Center, Wuhu, China

**Keywords:** authoritative parenting, cross-sectional study, infants and toddlers, parenting styles, weight-for-length z scores

## Abstract

**Objective:**

To explore the association between parenting styles among parents of infants and toddlers aged 0–36 months in China, and weight-for-length z scores (WFL-Z).

**Methods:**

This cross-sectional study recruited 1,164 eligible infants and toddlers (0–36 months) from July 2023 to March 2024 in China; 1,128 were included in the final analysis. Five parenting styles (indulgent, authoritative, inconsistent, authoritarian, permissive) were assessed using a standardized questionnaire. WFL-Z was calculated based on WHO growth standards. Multivariable linear regression models with progressive adjustment were applied, along with sex-stratified, age-stratified, and sensitivity analyses.

**Results:**

No statistically significant association was observed between parenting styles and WFL-Z in the primary analysis. Authoritative parenting was not significantly associated with WFL-Z (*B* = 0.006; *P* = 0.056). In sensitivity analyses, authoritative parenting was associated with a slightly increased likelihood of overweight (aOR = 1.019; *P* = 0.035); however, the effect size was minimal. Age-stratified analyses showed small and inconsistent associations, with limited significant findings observed in specific age groups.

**Conclusion:**

This study did not find robust evidence supporting an association between parenting styles and WFL-Z in early childhood. Findings from sensitivity and subgroup analyses were small in magnitude. Further longitudinal studies incorporating feeding-specific behaviors are needed to clarify potential relationships.

## Introduction

Childhood obesity is a major global public health challenge. World Health Organization estimates that about 35 million children under five worldwide are overweight, accounting for 5% of this age group (1). This proportion has increased steadily since 2000 across both high-income and low- and middle-income countries, with particularly rapid growth in East Asia (2). National surveys in China similarly show rising childhood overweight in recent years (3).

Early life—particularly the first 0–36 months—is recognized as a critical window for “programming” of weight trajectories (4). Elevated weight-for-length z scores (WFL-Z) and rapid weight gain during this period are associated with a higher risk of obesity and adverse metabolic outcomes later in childhood (5). Consequently, monitoring WFL-Z levels and their associated factors during infancy and early childhood is particularly important.

Parenting style reflects parents' attitudes, emotional climate, and behavioral control in childrearing (6). Yang's Parenting Style Questionnaire classifies parenting into five types: indulgent, authoritative, inconsistent, authoritarian, permissive (7). Parenting styles may be related to children's weight trajectories potentially through pathways such as eating behaviors, self-regulation, and physical activity (8, 9). Authoritative parenting (high responsiveness, moderate control) has been linked to healthier dietary patterns and stronger self-regulation (10). Whereas authoritarian or indulgent parenting is associated with more coercive or emotion-related feeding practices (e.g., pressure to eat, feeding in response to emotions), which have been associated excess intake and weight gain in some studies (11).

Evidence linking parenting styles with childhood overweight/obesity remains inconsistent. Some studies report that authoritative parenting is associated with lower BMI and healthier eating (12, 13); while others suggest that in East Asian contexts, controlling practices may show complex or indirect associations that differ from Western findings (14). A meta-analysis concluded that consistent associations have not been established and that many studies have high risk of bias, yielding low-quality evidence (15), Moreover, research has largely focused on school-age children and adolescents (16, 17), with limited evidence for infants and toddlers aged 0–36 months, particularly in non-Western settings such as China.

Therefore, this cross-sectional study examined infants and toddlers aged 0–36 months in urban Chinese communities to evaluate associations between parenting styles and WFL-Z, adjusting for sociodemographic and lifestyle confounders using multiple linear regression. We hypothesized that authoritative parenting might be associated with lower WFL-Z and reduced overweight risk, and that associations might differ by child sex. This study provides standardized cross-sectional evidence on parenting styles and weight status in early life within the cultural context of urban China.

## Methods

### Study population

**Figure 1:**
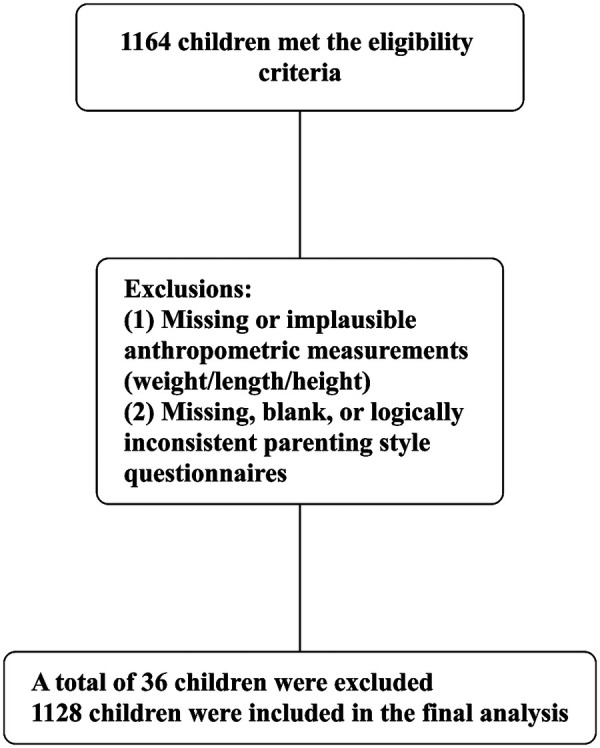
Flow chart of the study participants. Children refer to infants and toddlers aged 0–36 months.

This cross-sectional study was conducted from July 2023 to March 2024 in selected urban and suburban community health service centers in China, using convenience sampling of consecutive infants and toddlers (0–36 months) attending routine health examinations. Eligible participants were children whose parents provided written informed consent and completed an electronic questionnaire; children with major congenital, genetic, chronic conditions, illnesses affecting growth, invalid anthropometric data precluding WFL-Z calculation, or incomplete/invalid questionnaires were excluded. Of 1,164 eligible children, 1,128 were included in the final analysis. The study design and participant selection process are shown in Figure 1.

### Data collection

Data were obtained from (1) routine pediatric health examination records at participating community health service centers (e.g., weight and length/height) and (2) structured electronic questionnaires completed on-site by parents with researcher assistance, covering child characteristics, pregnancy-related factors, lifestyle and environmental factors, and parenting styles. Investigators checked questionnaires for completeness and basic logic at submission, and data underwent centralized quality control (range/consistency/outlier checks) with follow-up verification when needed; unresolvable records were excluded. Anthropometric assessors were blinded to questionnaire responses, and all data were de-identified prior to analysis.

### Variables

Exposure: Parenting style was assessed using the Parenting Style Questionnaire (7), which includes five dimensions (indulgent, authoritative, inconsistent, authoritarian, and permissive). Items are rated on a 5-point Likert scale (1 = never to 5 = always); dimension scores are summed, with higher scores indicating stronger traits. Test–retest reliability was 0.825 and the questionnaire has demonstrated good reliability in prior studies (18). For analyses, each dimension score was standardized and modeled as a continuous exposure.

Outcome: WFL-Z was calculated from anthropometric measurements using the WHO Child Growth Standards (19). WFL-Z was analyzed as a continuous outcome in the main analyses; for group comparisons and logistic regression, overweight (including obesity) was defined as WFL-Z > + 2 SD vs. non-overweight (WFL-Z ≤ + 2 SD). Anthropometric data were double-checked by two staff members blinded to questionnaire results.

Covariates were selected *a priori* based on prior literature and biological plausibility, including child, parental, pregnancy-related, lifestyle and environmental factors (e.g., age and sex, preterm birth, birth weight, parental BMI, cesarean delivery, night-time sleep duration, daily screen time, daily outdoor activity time, and second-hand smoke exposure). Continuous variables were modeled using raw values, and categorical variables were grouped according to the research question.

#### Daily screen time and daily outdoor activity time

According to guidelines and previous research, daily screen time as an ordinal categorical variable, categorized into four levels: none, <1 h, 1–2 h, and >2 h per day (20, 21). Daily outdoor activity time as an ordinal categorical variable, categorized into four levels: <1 h/day, 1–2 h/day, 2–3 h/day, and >3 h/day (22, 23).

### Data analysis

All analyses were performed using SPSS version 26.0 (IBM, Armonk, NY, USA), with a two-tailed *P* < 0.05 considered statistically significant. Internal consistency of each parenting style was assessed using Cronbach's alpha. Continuous variables are presented as mean ± SD for approximately normally distributed variables or median (IQR) for skewed variables, and categorical variables as *n* (%). Baseline characteristics were compared between non-overweight (WFL-Z ≤ + 2 SD) and overweight (including obesity; WFL-Z > + 2 SD) groups using independent-samples *t*-tests, Mann–Whitney *U* tests, or chi-square/Fisher's exact tests, as appropriate.

WFL-Z was analyzed as a continuous outcome using multivariable linear regression to examine associations with five parenting style scores (indulgent, authoritative, inconsistent, authoritarian, and permissive). Three models were fitted: Model 1 unadjusted; Model 2 adjusted for child characteristics; and Model 3 further adjusted for parental characteristics, pregnancy-related factors and lifestyle and environmental factors. Sex-stratified analyses and a parenting style × sex interaction term were used to assess effect modification. Sensitivity analyses used logistic regression with overweight (yes/no) as the outcome and additionally modeled authoritative parenting scores in tertiles to assess potential dose–response. Given the limited number of overweight cases, the events-per-variable ratio in the fully adjusted logistic regression model was relatively low, and these analyses were considered exploratory. Multicollinearity was evaluated using the variance inflation factor (VIF), with VIF < 5 indicating acceptable collinearity. Linear regression assumptions were assessed using residual diagnostics, and no major violations were observed. To address potential heterogeneity across age, additional analyses were conducted stratified by age groups (0–12, 13–24, and 25–36 months).

### Ethical statement

This study was conducted in accordance with the Declaration of Helsinki. The study protocol was reviewed and approved by an institutional ethics committee. Written informed consent was obtained from the parents or legal guardians of all participating infants and toddlers prior to enrollment. Participant information was de-identified before analysis.

## Results

Among the 1,164 children meeting inclusion criteria, 36 were excluded, resulting in 1,128 children included in the final analysis. The baseline characteristics of the included children are presented in Table 1. All parenting style scales demonstrated acceptable to excellent internal consistency (Cronbach's *α*s = 0.848–0.966; Indulgent = 0.852, Authoritative = 0.966, Permissive = 0.880, Authoritarian = 0.848, Inconsistent = 0.899).

**Table 1:** Baseline characteristics of the study population (*n* = 1,128).

Characteristic	Value
Child characteristics	
Age, months, mean ± SD	17.28 ± 10.91
Sex, male, *n* (%)	622 (55.1)
Length, cm, mean ± SD	80.45 ± 12.28
Weight, kg, mean ± SD	11.09 ± 2.99
WFL-Z, mean ± SD	0.62 ± 1.21
Preterm birth, *n* (%)	137 (12.1)
Birth weight, g, mean ± SD	3,235.14 ± 517.66
Parental characteristics	
Paternal age, years, mean ± SD	32.02 ± 4.74
Paternal BMI, kg/m^2^, mean ± SD	23.61 ± 3.12
Maternal age, years, mean ± SD	30.46 ± 4.52
Maternal BMI, kg/m^2^, mean ± SD	22.12 ± 3.11
Pregnancy-related factors	
Pregnancy complications, *n* (%)	230 (20.4)
Use of any nutritional supplement during pregnancy, *n* (%)	1,110 (98.4)
Folic acid supplementation, *n* (%)	1,021 (90.5)
Calcium supplementation, *n* (%)	579 (51.3)
Iron supplementation, *n* (%)	343 (30.4)
Vitamin A supplementation, *n* (%)	131 (11.6)
Vitamin D supplementation, *n* (%)	199 (17.7)
Progesterone use, *n* (%)	178 (15.8)
Firstborn child, *n* (%)	647 (57.4)
Cesarean delivery, *n* (%)	522 (46.3)
Lifestyle and environmental factors	
Daily screen time	
0 h/day, *n* (%)	635 (56.3)
<1 h/day, *n* (%)	299 (26.5)
1–2 h/day, *n* (%)	132 (11.7)
>2 h/day, *n* (%)	62 (5.5)
Daily outdoor activity time	
<1 h/day, *n* (%)	277 (24.6)
1–2 h/day, *n* (%)	456 (40.4)
2–3 h/day, *n* (%)	228 (20.2)
>3 h/day, *n* (%)	167 (14.8)
Night-time sleep duration, h, mean ± SD	10.54 ± 1.08
Second-hand smoke exposure, *n* (%)	203 (18.0)
Parenting style scores^a^	
Indulgent parenting, median [IQR]	8 [7, 11]
Authoritative parenting, mean ± SD	30.35 ± 13.04
Inconsistent parenting, median [IQR]	8 [6, 12]
Authoritarian parenting, mean ± SD	13.84 ± 5.57
Permissive parenting, median [IQR]	11.5 [9, 16]

Values are presented as mean ± SD or median (IQR) for continuous variables and as *n* (%) for categorical variables. WFL-Z was calculated using WHO Child Growth Standards. Preterm birth was defined as gestational age < 37 weeks. No missing data were observed for variables shown.

BMI, body mass index; WFL-Z, weight-for-length z score; WHO, World Health Organization; IQR, interquartile range.

_a_ Parenting style scores were assessed using the original scale; subscale names follow the scale terminology.

### Baseline characteristics of study participants

A total of 1,128 children aged 0–36 months were included in the final analysis. The mean age was 17.28 ± 10.91 months, 55.1% were boys, and the mean WFL-Z was 0.62 ± 1.21. Overall, 9.4% of children were classified as overweight (including obesity) (Table 1).

### Univariate analysis by WFL-Z group

Univariate analysis by WFL-Z group revealed that children in the overweight group were younger and had longer night-time sleep duration (all *P* < 0.05), while differences in sex and parental BMI did not reach statistical significance (Supplementary Table S1).

### Multiple linear regression analysis model

As shown in Table 2, three hierarchical linear regression models were fitted with WFL-Z as the dependent variable. In Model 1, inconsistent parenting score was inversely associated with WFL-Z (*B* = −0.032, 95% CI: −0.063 to −0.001; *P* = 0.048), whereas other parenting style scores were not significant. After adjustment for age, sex, preterm birth, and birth weight in Model 2, authoritative parenting score was positively associated with WFL-Z (*B* = 0.006, 95% CI: 0.000–0.012; *P* = 0.043). In the fully adjusted Model 3, authoritative parenting was not significantly associated with WFL-Z (*B* = 0.006, 95% CI: 0.000–0.012; *P* = 0.056), and no other parenting style scores showed significant associations (Table 2).

**Table 2:** Multivariable linear regression of parenting style scores and children's WFL-Z.

Variables	Model 1 *B* (95% CI), *P*	Model 2 *B* (95% CI), *P*	Model 3 *B* (95% CI), *P*
Indulgent parenting score	0.012 (−0.013, 0.037), 0.389	0.020 (−0.005, 0.045), 0.123	0.018 (−0.008, 0.043), 0.171
Authoritative parenting score	0.005 (−0.001, 0.011), 0.128	0.006 (0.000, 0.012), 0.043	0.006 (0.000, 0.012), 0.056
Inconsistent parenting score	−0.032 (−0.063, −0.001), 0.048	−0.026 (−0.057, 0.005), 0.095	−0.025 (−0.057, 0.006), 0.118
Authoritarian parenting score	0.006 (−0.018, 0.030), 0.635	0.006 (−0.016, 0.028), 0.575	0.006 (−0.016, 0.028), 0.573
Permissive parenting score	−0.001 (−0.023, 0.021), 0.898	−0.004 (−0.026, 0.018), 0.683	−0.004 (−0.026, 0.018), 0.689
Age (months)	–	−0.023 (−0.029, −0.017), <0.001	−0.022 (−0.030, −0.014), <0.001
Sex (male vs. female)	–	0.199 (0.062, 0.336), 0.005	0.212 (0.073, 0.351), 0.003
Preterm birth	–	0.382 (0.157, 0.607), 0.001	0.380 (0.153, 0.607), 0.001
Birth weight (per 1 kg)	–	0.374 (0.232, 0.517), <0.001	0.372 (0.228, 0.517), <0.001

Values are unstandardized regression coefficients (*B*) with 95% confidence intervals (CI) and *P* values from linear regression models; the dependent variable was WFL-Z. *B* represents the mean change in WFL-Z per 1-unit increase in the predictor (e.g., per 1-point increase in parenting style score). “–” indicates the variable was not included in the model.

Model 1: Included the five parenting style scores only.

Model 2: Additionally adjusted for child age, sex, preterm birth, and birth weight.

Model 3: Adjusted for all covariates, including child characteristics,parental characteristics, pregnancy-related factors and lifestyle/environmental factors.

### Gender stratified analysis

After stratification by gender and adjustment for confounding factors, the linear association between authoritative parenting style and WFL-Z did not reach statistical significance in either boys or girls (both *P* > 0.05) (Supplementary Table S2).

### Age-stratified analysis

Age-stratified analyses are presented in Supplementary Table S3. In the 0–12 months group, authoritarian parenting was positively associated with WFL-Z (*B* = 0.040, *P* = 0.037), whereas no other parenting styles were significant. No significant associations were observed in the 13–24 months group (all *P* > 0.05). In the 25–36 months group, indulgent parenting was positively associated with WFL-Z (*B* = 0.048, *P* = 0.038), while other parenting styles remained non-significant. Overall, the observed associations were small in magnitude and not consistent across age groups.

### Sensitivity analysis 1: binary logistic regression

After full adjustment, authoritative parenting score and night-time sleep duration were positively associated with overweight, whereas child age and preterm birth were inversely associated (all *P* < 0.05). Prenatal nutrient supplementation was also associated with higher odds of overweight (*P* = 0.044); other covariates were not significant (Table 3).

**Table 3:** Fully adjusted multivariable logistic regression for childhood overweight.

Variables	aOR (95% CI)	*P* value
Parenting style		
Authoritative parenting score	1.019 (1.001, 1.037)	0.035
Child characteristics		
Age (months)	0.963 (0.936, 0.990)	0.007
Sex (male)	0.676 (0.440, 1.040)	0.075
Preterm birth	0.475 (0.262, 0.858)	0.014
Birth weight (per 1 kg)	1.484 (0.965, 2.284)	0.073
Parental characteristics		
Paternal age (years)	1.033 (0.970, 1.100)	0.314
Paternal BMI (kg/m^2^)	1.020 (0.955, 1.089)	0.552
Maternal age (years)	1.006 (0.938, 1.080)	0.860
Maternal BMI (kg/m^2^)	0.995 (0.930, 1.065)	0.892
Pregnancy-related factors		
Pregnancy complications	1.410 (0.815, 2.441)	0.219
Use of any nutritional supplement during pregnancy	3.442 (1.032, 11.478)	0.044
Firstborn child	0.736 (0.456, 1.188)	0.209
Cesarean delivery	1.510 (0.968, 2.354)	0.069
Lifestyle and environmental factors		
Daily screen time		
Reference group	1.00 (Ref)	
<1 h/day	0.654 (0.244, 1.757)	0.400
1–2 h/day	0.679 (0.254, 1.817)	0.441
>2 h/day	0.868 (0.296, 2.544)	0.796
Daily outdoor activity time		
<1 h/day (Ref)	1.00 (Ref)	
1–2 h/day	1.260 (0.615, 2.581)	0.527
2–3 h/day	0.837 (0.430, 1.628)	0.600
>3 h/day	0.809 (0.386, 1.695)	0.574
Night-time sleep duration (h/night)	1.281 (1.067, 1.537)	0.008
Second-hand smoke exposure	0.944 (0.544, 1.636)	0.837

aOR, adjusted odds ratio; CI, confidence interval; BMI, body mass index; Ref, reference category.

The dependent variable was overweight (yes vs. no). The model included authoritative parenting score; child characteristics; parental characteristics; pregnancy-related factors and lifestyle and environmental factors. Birth weight was entered as a continuous variable per 1 kg increase.

### Sensitivity analysis 2: subgroup analysis

No significant linear trend was observed across tertiles. Compared with the low authoritative parenting group, the high group showed a slightly higher adjusted WFL-Z (*B* = 0.181, 95% CI: 0.003–0.358; *P* = 0.046), whereas no difference was observed for the medium group (*B* = −0.053, 95% CI: −0.229 to 0.123; *P* = 0.552) (Table 4).

**Table 4:** Association between tertiles of authoritative parenting score and children WFL-Z: multivariable linear regression.

Authoritative parenting score group	*B* (95% CI)	*P* value
Low	Reference	–
Medium	−0.053 (−0.229, 0.123)	0.552
High	0.181 (0.003, 0.358)	0.046
*P* for trend		0.148

CI, confidence interval.

*B* coefficients and 95% CIs were estimated from multivariable linear regression models with WFL-Z as the dependent variable. The low tertile of authoritative parenting score was used as the reference group.

## Discussion

In the main analysis, we did not observe a statistically significant association between parenting style and infant WFL-Z. These findings do not support a clear or consistent relationship between parenting style and early growth in this population. In sensitivity analyses, prenatal nutritional supplementation was associated with higher odds of overweight; however, this result should be interpreted with caution, as the estimate may be unstable and influenced by residual confounding and the high prevalence of supplementation in this sample. One possible explanation is that the influence of parenting style on weight-related outcomes may be indirect and less pronounced during early infancy. At this stage, infant growth is strongly driven by biological factors and immediate feeding practices (24–26), which may attenuate or obscure potential effects of broader parenting patterns. In addition, parenting style may exert its influence through pathways such as feeding behaviors, emotional regulation, or activity patterns, which were not directly captured in the present analysis.Existing research has more frequently focused on feeding practices or dietary patterns rather than broader parenting styles when examining early-life weight outcomes (27, 28). While some studies have suggested that parental behaviors related to feeding may be associated with infant growth (29), evidence regarding overall parenting style remains limited and inconclusive. The present findings are therefore consistent with the current lack of clear evidence in this area.

Due to the cross-sectional design, causal direction cannot be established. It is possible that infant weight may be associated with differences in parental responses, suggesting a potential bidirectional relationship, as noted in previous studies (30). This should be further examined in longitudinal research.

Several limitations should be considered. The cross-sectional design precludes causal inference, and participants were recruited through convenience sampling from community health service centers in China, with a relatively high prevalence of prenatal nutritional supplementation, which may limit generalizability. In addition, parenting style was assessed by parental self-report using a general instrument not specifically developed for very young infants, and detailed feeding-related variables were not collected, which may introduce measurement error, reporting bias, and residual confounding. Finally, multiple comparisons were conducted across parenting dimensions and subgroup analyses, which may increase the risk of type I error.

Despite these limitations, this study contributes to the literature by examining parenting style in relation to infant weight outcomes using a validated parenting assessment tool in a relatively understudied population. The findings highlight the need for further research using longitudinal designs and more comprehensive assessment of feeding-related and behavioral pathways through which parenting may influence early growth trajectories.

## Conclusion

In conclusion, this study did not find evidence of a statistically significant association between parenting style and infant weight-for-length z-scores. The overall pattern of results, including sensitivity and subgroup analyses, does not support a consistent or robust relationship. Further research using longitudinal designs and more detailed assessments of feeding-related behaviors may help clarify these associations.

## Data Availability

The raw data supporting the conclusions of this article will be made available by the authors, without undue reservation.
