# Supplementary material for: Association between parenting styles and weight-for-length z scores among infants and toddlers aged 0–36 months in China: a cross-sectional study
Source: Front Pediatr. 2026 May 20;14:1827938. doi: 10.3389/fped.2026.1827938 (PMC13229880; doi:10.3389/fped.2026.1827938)
Supplement: Supplementary file 1 [file Supplementaryfile1.docx]

| **variables** | **WFL-Z ≤ +2 SD（n=1022）** | **WFL-Z > +2 SD（n=106）** | ***t/*χ^2^*/z/H*** | ***P*** |
| --- | --- | --- | --- | --- |
| **Child characteristics** | | | | |
| Age (months) | 17.63 ± 10.89 | 13.9 ± 10.55 | 3.370 | 0.001 |
| Sex (male) | 556（54.4%） | 66（62.3%） | 2.399 | 0.121 |
| Length(cm) | 80.90 ± 12.17 | 76.11 ± 12.53 | 3.846 | 0.000 |
| Weight(kg) | 10.94 ± 2.92 | 12.39 ± 3.39 | 4.779 | 0.000 |
| Preterm birth | 118（11.5%） | 19（17.9%） | 3.662 | 0.056 |
| Birth weight(g) | 3228.13 ± 509.19 | 3302.73 ± 591.62 | 1.413 | 0.158 |
| **Parental characteristics** | | | | |
| Paternal age(years) | 32.02 ± 4.79 | 32.08 ± 4.28 | 0.137 | 0.891 |
| Paternal BMI(kg/m²) | 23.58 ± 3.13 | 23.88 ± 3.02 | 0.935 | 0.350 |
| Maternal age( years) | 30.47 ± 4.53 | 30.31 ± 4.43 | 0.345 | 0.730 |
| Maternal BMI(kg/m²) | 22.12 ± 3.12 | 22.14 ± 3.02 | 0.062 | 0.950 |
| **Pregnancy‑related factors** | | | | |
| Pregnancy complications, n (%) | 211（20.6%） | 19（17.9%） | 0.438 | 0.508 |
| Use of any nutritional supplement during pregnancy, n (%) | 1008（98.6%） | 102（96.2%） | / | 0.080 |
| Firstborn child, n (%) | 582（56.9%） | 65（61.3%） | 0.751 | 0.386 |
| Cesarean delivery, n (%) | 479（46.9%） | 43（40.6%） | 1.535 | 0.215 |
| **Lifestyle and environmental factors** | | | | |
| Daily screen time |  |  |  |  |
| 0 h/day, n (%) | 569（55.7%） | 66（62.3%） | *H*=1.257 | 0.262 |
| <1 h/day, n (%) | 276（21.7%） | 23（21.7%） |  |  |
| 1–2 h/day, n (%) | 121（11.8%） | 11（10.4%） |  |  |
| >2 h/day, n (%) | 56（5.5%） | 6（5.7%） |  |  |
| Daily outdoor activity time |  |  |  |  |
| ＜1 h/day, n (%) | 239（23.4%） | 38（35.8%） | *H*=3.555 | 0.059 |
| 1–2 h/day, n (%) | 421（41.2%） | 35（33%） |  |  |
| 2–3 h/day, n (%) | 211（20.6%） | 17（16%） |  |  |
| >3 h/day, n (%) | 151（14.8%） | 16（15.1%） |  |  |
| Night‑time sleep duration(h) | 10.51 ± 1.04 | 10.84 ± 1.35 | 2.40 | 0.018 |
| Second‑hand smoke exposure, n (%) | 184（18%） | 19（17.9%） | 0.000 | 0.984 |
| **Parenting style scores*** | | | | |
| Indulgent parenting score | 8[7，11] | 8[7，12] | -0.738 | 0.461 |
| Authoritative parenting score | 34[19，40] | 37.5[24，44] | -2.240 | 0.025 |
| Inconsistent parenting score | 8[6，12] | 8[6，12] | -0.766 | 0.444 |
| Authoritarian parenting score | 13.84 ± 5.60 | 13.08 ± 5.31 | 0.071 | 0.943 |
| Permissive parenting score | 12[9，16] | 11[9，16] | -0.397 | 0.691 |

Supplementary Table S1. Univariable analysis by WFL‑Z group (WFL‑Z ≤ +2 SD vs > +2 SD).

Note: Continuous variables are presented as mean ± standard deviation (SD), and categorical variables as n (%). *t/*χ^2^*/z/H* denote the corresponding test statistics. Age, length, weight, paternal and maternal age, and BMI were compared using the independent-samples *t* test. Categorical variables were compared using the chi-square test. Non-normally distributed continuous variables and ordinal variables were compared using rank-sum tests. *P* < 0.05 was considered statistically significant.

Abbreviations: WFL-Z, weight-for-length z score; BMI, body mass index; SD, standard deviation. χ^2^, chi-square; *z*, standardized statistic from Mann-Whitney *U* test; H, Kruskal-Wallis test statistic.

| **Domain** | **Variable (unit)** | **Category** | **Boys, *β* (95% CI)** | ***P*** | **Girls, *β* (95% CI)** | ***P*** |
| --- | --- | --- | --- | --- | --- | --- |
| **Child characteristics** | Age (months) | per 1 month | -0.023 (-0.033, -0.011) | <0.001 | -0.019 (-0.033, -0.005) | 0.007 |
|  | Preterm birth | Yes vs No | 0.522 (0.222, 0.822) | <0.001 | 0.286 (-0.068, 0.641) | 0.113 |
|  | Birth weight (g)* | per 1 g | 4×10^-4^  (2×10^-4^ ,5×10^-4^) | <0.001 | 4×10^-4^  (2×10^-4^ ,6×10^-4^) | 0.001 |
| **Parental characteristics** | Paternal BMI (kg/m²) | per 1 kg/m² | 0.010 (-0.018, 0.038) | 0.464 | 0.004 (-0.033, 0.041) | 0.837 |
|  | Paternal age (years) | per 1 year | -0.006 (-0.032, 0.021) | 0.685 | 0.018 (-0.021, 0.056) | 0.375 |
|  | Maternal BMI (kg/m²) | per 1 kg/m² | 0.014 (-0.014, 0.043) | 0.317 | 0.003 (-0.035, 0.040) | 0.894 |
|  | Maternal age (years) | per 1 year | 0.022 (-0.007, 0.051) | 0.133 | -0.029 (-0.070, 0.012) | 0.161 |
| **Pregnancy-related factors** | Pregnancy complications | Yes vs No | -0.101 (-0.322, 0.120) | 0.371 | 0.009 (-0.276, 0.296) | 0.952 |
|  | Use of any nutritional supplement during pregnancy | Yes vs No | 0.365 (-0.333, 1.063) | 0.305 | -0.384 (-1.289, 0.522) | 0.406 |
|  | Firstborn child | Yes vs No | 0.003 (-0.193, 0.198) | 0.977 | 0.022 (-0.241, 0.284) | 0.872 |
|  | Cesarean delivery | Yes vs No | -0.038 (-0.220, 0.143) | 0.679 | 0.040 (-0.201, 0.281) | 0.745 |
| **Lifestyle and environmental factors** | Daily screen time | 0 h | Ref | — | Ref | — |
|  |  | <1 h vs 0 h | -0.010 (-0.246, 0.228) | 0.936 | -0.119 (-0.424, 0.185) | 0.442 |
|  |  | 1–2 h vs 0 h | 0.021 (-0.291, 0.334) | 0.893 | -0.135 (-0.581, 0.311) | 0.552 |
|  |  | >2 h vs 0 h | 0.198 (-0.234, 0.629) | 0.368 | 0.123 (-0.428, 0.674) | 0.661 |
|  | Daily outdoor activity time | <1 h | Ref | — | Ref | — |
|  |  | 1–2 h vs <1 h | 0.168 (-0.074, 0.410) | 0.174 | -0.392 (-0.701, -0.083) | 0.013 |
|  |  | 2–3 h vs <1 h | 0.040 (-0.246, 0.325) | 0.786 | -0.257 (-0.625, 0.110) | 0.170 |
|  |  | >3 h vs <1 h | 0.103 (-0.216, 0.422) | 0.525 | -0.245 (-0.657, 0.167) | 0.243 |
|  | Night-time sleep duration(h) | per 1 h | -0.009 (-0.091, 0.073) | 0.835 | 0.118 (0.011, 0.226) | 0.031 |
|  | Second-hand smoke exposure | Yes vs No | 0.019 (-0.210, 0.248) | 0.874 | -0.085 (-0.396, 0.225) | 0.589 |
| **Parenting style scores** | Indulgent parenting score | per 1 point | 0.027 (-0.005, 0.059) | 0.099 | 0.007 (-0.036, 0.051) | 0.737 |
|  | Authoritative parenting score | per 1 point | 0.007 (-0.001, 0.015) | 0.094 | 0.003 (-0.008, 0.013) | 0.619 |
|  | Inconsistent parenting score | per 1 point | -0.014 (-0.052, 0.024) | 0.464 | -0.034 (-0.088, 0.019) | 0.209 |
|  | Authoritarian parenting score | per 1 point | -0.007 (-0.035, 0.021) | 0.618 | 0.030 (-0.008, 0.068) | 0.120 |
|  | Permissive parenting score | per 1 point | -0.018 (-0.0450, 0.008) | 0.179 | 0.003 (-0.033, 0.040) | 0.857 |

Supplementary Table S2. Sex-stratified multivariable linear regression for WFL-Z

Note: Values are regression coefficients (*β*) with 95% confidence intervals (CIs) from sex-stratified multivariable linear regression models. *β* represents the change in WFL-Z per 1-unit increase in the predictor (continuous variables) or relative to the reference category (categorical variables). “Ref” indicates the reference group. *P* values are two-sided; *P* <0.001 is reported as “<0.001”. Birth weight is modeled per 1 g and therefore reported in scientific notation. Abbreviations: WFL-Z, weight-for-length z score; BMI, body mass index; CI, confidence interval.

| **parenting scores** | **0-12 months *B*** | **0-12 months *P*** | **13-24 months *B*** | **13-24 months *P*** | **25-36 months *B*** | **25-36 months *P*** |
| --- | --- | --- | --- | --- | --- | --- |
| Indulgent parenting score | 0.013 | 0.577 | 0.011 | 0.614 | 0.048 | 0.038^*^ |
| Authoritative parenting score | 0.009 | 0.07 | 0.009 | 0.121 | -0.009 | 0.249 |
| Inconsistent parenting score | -0.015 | 0.598 | -0.025 | 0.336 | -0.026 | 0.353 |
| Authoritarian parenting score | 0.04 | 0.037^*^ | -0.03 | 0.093 | 0.004 | 0.849 |
| Permissive parenting score | -0.03 | 0.123 | 0.018 | 0.332 | -0.007 | 0.719 |

Supplementary Table S3. Associations between parenting styles and WFL-Z stratified by age groups

Note: abbreviations: *B*, unstandardized regression coefficient. Models were adjusted for all covariates, including child characteristics; parental characteristics; pregnancy-related factors and lifestyle/environmental factors.
